# The cost-effectiveness of germline BRCA testing-guided olaparib treatment in metastatic castration resistant prostate cancer

**DOI:** 10.1017/S0266462324000011

**Published:** 2024-03-05

**Authors:** Srinivas Teppala, Paul A. Scuffham, Haitham Tuffaha

**Affiliations:** 1 Centre for Applied Health Economics, Griffith University, Nathan, QLD, Australia; 2 Menzies Health Institute Queensland, Griffith University, Southport, QLD, Australia; 3 Centre for the Business and Economics of Health, The University of Queensland, St. Lucia, QLD, Australia

**Keywords:** germline testing, BRCA, metastatic castration resistant prostate cancer, cost effectiveness, poly-adenosine diphosphate ribose polymerase (PARP) inhibitors, olaparib

## Abstract

**Background:**

Olaparib targets the DNA repair pathways and has revolutionized the management of metastatic castration resistant prostate cancer (mCRPC). Treatment with the drug should be guided by genetic testing; however, published economic evaluations did not consider olaparib and genetic testing as codependent technologies. This study aims to assess the cost-effectiveness of *BRCA* germline testing to inform olaparib treatment in mCRPC.

**Methods:**

We conducted a cost-utility analysis of germline *BRCA* testing-guided olaparib treatment compared to standard care without testing from an Australian health payer perspective. The analysis applied a decision tree to indicate the germline testing or no testing strategy. A Markov multi-state transition approach was used for patients within each strategy. The model had a time horizon of 5 years. Costs and outcomes were discounted at an annual rate of 5 percent. Decision uncertainty was characterized using probabilistic and scenario analyses.

**Results:**

Compared to standard care, *BRCA* testing-guided olaparib treatment was associated with an incremental cost of AU$7,841 and a gain of 0.06 quality-adjusted life-years (QALYs). The incremental cost-effectiveness ratio (ICER) was AU$143,613 per QALY. The probability of *BRCA* testing-guided treatment being cost effective at a willingness-to-pay threshold of AU$100,000 per QALY was around 2 percent; however, the likelihood for cost-effectiveness increased to 66 percent if the price of olaparib was reduced by 30 percent.

**Conclusion:**

This is the first study to evaluate germline genetic testing and olaparib treatment as codependent technologies in mCRPC. Genetic testing-guided olaparib treatment may be cost-effective with significant discounts on olaparib pricing.

## Introduction

Prostate cancer is the most diagnosed non-skin cancer and second leading cancer-related cause of mortality in Australian men (1). Approximately 3,568 deaths due to prostate cancer were reported in 2020 (1). Between 4.6 and 17 percent of patients with prostate cancer have mutations in germline DNA-repair genes (2;3). The prevalence of pathogenic variants is substantially higher in metastatic prostate cancer (11.8 percent) (4) compared to local prostate cancer (4.6 percent) (5). In addition, more than half of mutations in metastatic prostate cancer are in the *BRCA* genes (*BRCA2*: 44 percent, *BRCA1*: 7 percent) (4).

Prostate cancer patients with *BRCA* mutations have a more aggressive form of disease with higher risk of nodal involvement, distant metastasis, and poor overall survival (6;7). Despite the grim prognosis, poly-adenosine diphosphate ribose polymerase (PARP) inhibitors have shown promising results in the treatment of metastatic castration resistant prostate cancer (mCRPC). These drugs are selective for homologous DNA repair mutations (8). The PARP inhibitors olaparib (9) and rucaparib (10) are FDA approved and recommended for *BRCA* positive mCRPC patients who had prior treatment with novel hormonal agents (NHAs) such as abiraterone or enzalutamide or taxanes (e.g., docetaxel and cabazitaxel). Rucaparib, however, is not TGA approved for use in Australia (11).

Three studies (12-14) have examined the cost-effectiveness of PARP therapy in mCRPC and there is considerable variation in the reported results. The study by Su et al. (13) showed that olaparib was cost-effective; however, the results from the other two studies (12;14) suggested that treatment with olaparib was not value for money. All three studies (12-14) considered the cost of testing but did not account for the codependent nature of olaparib therapy (i.e., overlooked the fact that treatment decision with the drug was conditional on testing results). Additionally, some of the studies used a partitioned modeling approach which has several limitations including, the inability to account for transitions between health states, reliance on proxy measures to define health states, failure to account for the dependence of survival on effects from other treatments, and an overall tendency for poor predictability beyond the trial period (15). Also, the study by Xu et al. (14) indicated that the cost-effectiveness of olaparib could vary based on the country or setting. In summary, existing studies (12-14) relied heavily on data from the PROfound trial (9), used partitioned modeling (13) and did not consider the companion nature or dependence of treatment decisions on genetic testing. Given these shortcomings, we aim to evaluate the cost-effectiveness of olaparib therapy in mCRPC compared to the standard care alternative (16) from an Australian health system perspective, after considering the codependent nature of the technologies (*BRCA* testing and olaparib treatment) and utilizing a state transition modeling approach.

## Materials and methods

### Model description

The model evaluated the cost-effectiveness of germline testing for *BRCA* variants (BRCA1 and BRCA2) to inform olaparib treatment in a hypothetical cohort of men with mCRPC, who had disease progression while receiving first-line treatment with a NHA, abiraterone or enzalutamide. Disease progression in the cohort signifies, biochemical (i.e., three consecutive rises of prostate specific antigen, 1 week apart with a 50 percent increase over the nadir or a prostate specific antigen level > 2 ng/ml) or radiological progression (i.e., appearance of two or more bone lesions) while having castrate levels of testosterone (i.e., levels <50 ng/dl or 1.7 nmol/l). A decision tree of germline *BRCA* testing versus no testing followed by a Markov multi-health state transition model was developed using TreeAge Pro (TreeAge Software, Williamstown, MA, USA) (17).

**Figure 1.**
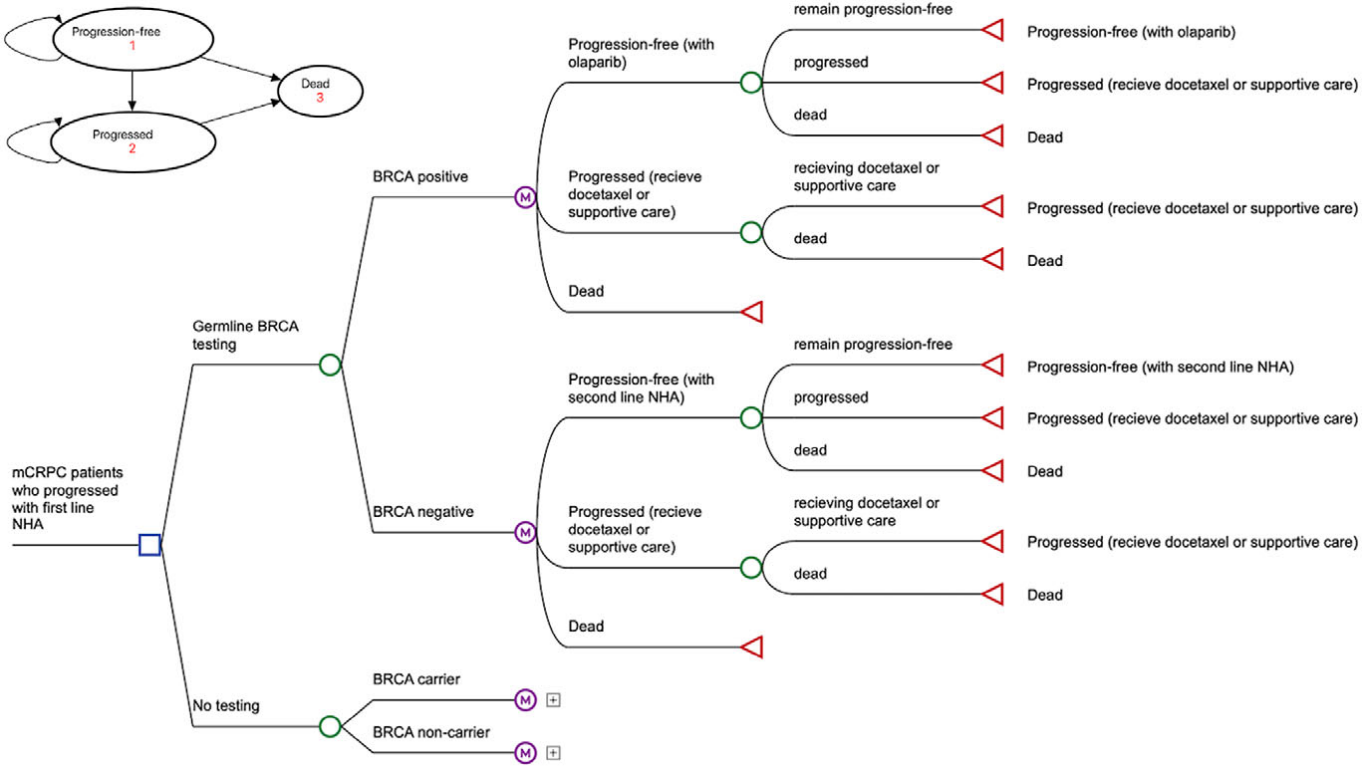
Schematic of the model.

The structure of the model is presented in Figure 1. All hypothetical patients in the cohort are eligible for germline testing (18). The *BRCA* positive patients receive treatment with the PARP inhibitor olaparib. Upon treatment patients were expected to be in one of three health states: progression-free, progressed disease or dead. Patients who progressed while on olaparib were assumed to receive subsequent treatment with docetaxel for a maximum of four cycles, and supportive care thereafter. The ceiling of 4 months for docetaxel was established from typical patterns of treatment with the drug in mCRPC patients (19;20). The choice for olaparib (as second-line treatment in *BRCA* positive patients) and docetaxel (third-line treatment) was based on practice recommendations in patients with mCRPC (18;21). The *BRCA* negative patients, as well as all patients in the no testing pathway were assumed to receive second-line treatment with a second NHA, that is, patients with prior treatment with abiraterone receive enzalutamide and vice versa (21). The proportion of patients receiving second-line enzalutamide (45 percent) or abiraterone (55 percent) was derived from the PROfound trial (9). Similar to *BRCA* positive patients with disease progression while on olaparib, patients on NHA in the comparator (i.e., no germline testing), were assumed to receive further treatment with docetaxel followed by supportive care. For simplification (i.e., similarity to the *BRCA* negative arm), the decision tree for the comparator has not been included in Figure 1. Please refer to Supplementary Material for a more detailed overview. The Markov health states (progression-free, progressed, and dead) for patients are similar in both the *BRCA* testing and no testing pathways and have been provided in the top-left corner of Figure 1.

### Model inputs

A summary of the parameters used in the model is provided in Table 1. The prevalence of *BRCA* variants in metastatic prostate cancer is variable (6–14 percent) (4;22). For our base case analysis, we chose a *BRCA* positive probability of 10 percent, based on a Medical Services Advisory Committee (MSAC) of Australia evaluation (23). The probabilities for disease progression while receiving olaparib were derived from a subset of *BRCA* positive patients receiving the drug in the PROfound trial (9). Given the maturity of the overall survival data (24), we did not perform extrapolation using alternative parametric distributions and instead used the exponential distribution to estimate transition probabilities (*S*[*t*] = *e* ^-λ*t*^; *S* = survival at time, *t*; λ = average number of events in time, *t*) (17). Disease progression with NHA in variant negative patients was modeled based on the study by Shore et al. (25). Carriers of *BRCA* variants typically have more aggressive disease progression (6). Therefore, despite the same treatment (i.e., second-line NHA), for all patients in the no testing strategy, we assumed faster disease progression in patients who harbor *BRCA* variants and as such used progression rates with NHA reported for this subset of the cohort from the PROfound trial (median = 3.0 months) (9). Background mortality rates were obtained from the Australian Bureau of Statistics (26). Mortality in patients on salvage therapy with docetaxel and supportive care was assumed to be similar across the two arms and was obtained from the study by Miyake et al. (27). The frequencies of serious adverse events (grade ≥ 3) in patients receiving treatment with NHA or olaparib was obtained from the PROfound trial (9), while results from the TAX 327 trial (20) were used for adverse events associated with docetaxel.

**Table 1.** Summary of parameters used in the model

Parameters	Mean/median value	Range	Distribution	Source
Clinical inputs				
PFS in variant-positive patients receiving olaparib, months	9.8	–	Exponential; λ = 0.0707	de Bono et al., 2020 (9)
PFS in variant-positive patients receiving NHA, months	3.0	–	Exponential; λ = 0.2310	de Bono et al., 2020 (9)
PFS in variant-negative patients receiving NHA, months	3.5	–	Exponential; λ = 0.1992	Shore et al., 2021 (25)
OS in patients receiving salvage therapy with docetaxel and supportive care, months	14.9	–	Exponential; λ = 0.0465	Miyake et al., 2021 (27)
Background annual mortality rate	0.01	–	Fixed	ABS, 2021 (26)
Proportion of BRCA positive patients	0.10	0.08–0.13	Beta; α = 53.15, β = 478.39	MSAC, 2021 (23)
Proportion of the control group receiving the NHA, abiraterone	0.55	0.41–0.69	Beta; α = 28.04, β = 22.94	de Bono et al., 2020 (9)
Proportion of the control group receiving the NHA, enzalutamide	0.45	0.34–0.56	Beta; α = 35.06, β = 42.86	de Bono et al., 2020 (9)
Proportion of patients with adverse events (grade ≥ 3) with olaparib				
Anemia, grade III	0.21	0.16–0.26	Beta; α = 51.33, β = 193.09	de Bono et al., 2020 (9)
Vomiting, grade III	0.01	0.009–0.2	Beta; α = 35.56, β = 2,927.44	de Bono et al., 2020 (9)
Back pain, grade III	0.008	0.006–0.01	Beta; α = 63.48, β = 7,871.52	de Bono et al., 2020 (9)
Fatigue, grade III	0.03	0.02–0.03	Beta; α = 78.79, β = 2,839.21	de Bono et al., 2020 (9)
Proportion of patients with adverse events (grade ≥ 3) with NHA				
Anemia, grade III	0.05	0.04–0.07	Beta; α = 56.24, β = 985.29	de Bono et al., 2020 (9)
Back pain, grade III	0.02	0.01–0.02	Beta; α = 55.39, β = 3,637.36	de Bono et al., 2020 (9)
Fatigue, grade III	0.05	0.04–0.07	Beta; α = 56.24, β = 985.29	de Bono et al., 2020 (9)
Proportion of patients with adverse events (grade ≥ 3) with docetaxel				
Anemia, grade III	0.02	0.01–0.02	Beta; α = 55.39, β = 3,637.36	Tannock et al., 2004 (20)
Vomiting, grade III	0.06	0.05–0.08	Beta; α = 52.82, β = 827.43	Lee et al., 2013 (66)
Fatigue, grade III	0.02	0.01–0.02	Beta; α = 55.39, β = 3,637.36	Tannock et al., 2004 (20)
Utility/disutility weights				
Progression-free while receiving NHA or olaparib	0.76	0.69–0.78	Beta; α = 358.05, β = 114.94	Clarke et al., 2022 (38)
Progression-free while receiving docetaxel	0.69	0.59–0.80	Beta; α = 51.85, β = 23.30	Lloyd et al., 2015 (39)
Supportive Care	0.37	0.33–0.41	Beta: α = 215.2, β = 366.5	Barqawi et al., 2019 (35)
Anemia, grade III	−0.12	−0.14 – −0.08	Beta: α = 55.33, β = 409.62	Barqawi et al., 2019 (35)
Back pain, grade III	−0.07	−0.08 – −0.05	Beta: α = 65.37, β = 910.36	Barqawi et al., 2019 (35)
Fatigue, grade III	−0.47	−0.59 – −0.36	Beta: α = 33.40, β = 37.21	Barqawi et al., 2019 (35)
Vomiting, grade III	−0.21	−0.25 – −0.17	Beta: α = 86.89, β = 326.86	Hall et al., 2019 (40)
Cost inputs in AU$*				
Genetic testing; using listed 75% discount pricing	750	563–938	Gamma; α = 64, λ =0.085	MBS item 73304 (28)
Cost of genetic counseling	283	212–354	Gamma; α = 64, λ =0.23	MBS item 132 (29)
Olaparib (300 mg twice daily) per month	6,631	4,973–8,288	Gamma; α = 64, λ = 0.01	PBS item 11528R (34)
Abiraterone (1,000 mg/day) + prednisone (10 mg/day) per month	3,227	2,421–4,034	Gamma; α = 64, λ = 0.019	PBS items 11206T (30) and 1935W (31)
Enzalutamide (160 mg/day) per month	3,537	2,653–4,421	Gamma; α = 64, λ = 0.018	PBS item 10174L (33)
Docetaxel (@75 mg/m^2^ every 3 weeks) + prednisone (@5 mg/day) per month	161	120–201	Gamma; α = 64, λ = 0.40	PBS items 10148D (32) and 1935W (31)
Costs of supportive care per day	242	182–303	Gamma; α = 64.01, λ = 0.26	Cronin et al., 2017 (67)
Costs of treating an anemia (grade ≥ 3) episode	1,602	1,442–1,763	Gamma; α = 399.24, λ = 0.25	Barqawi et al., 2019 (35)
Costs of treating a vomiting (grade ≥ 3) episode	23,526	19,997–27,055	Gamma; α = 64, λ = 0.003	Roeland et al., 2018 (36)
Costs of treating a back pain (grade ≥ 3) episode	17,705	15,934–19,475	Gamma; α = 400, λ = 0.022	Barqawi et al., 2019 (35)
Costs of treating a fatigue (grade ≥ 3) episode	13,924	12,950–14,899	Gamma; α = 817.43, λ = 0.059	Barqawi et al., 2019 (35)

* Converted to 2021 AU$ where necessary using the Campbell and Cochrane Economics Methods Group (CCEMG) cost converter (37).

### Costs

Costs for germline genetic testing (28), pre-and-post-test genetic counseling (29) were obtained from the Medicare Benefits Schedule (items 73304, 132). Treatment costs for the NHAs (abiraterone: 1,000 mg/day along with prednisone 5 mg/twice daily, AU$ 115/dose; enzalutamide: 160 mg/day, AU$ 126/dose), olaparib (300 mg/twice daily, AU$ 237 per dose), and docetaxel (75 mg/m^2^ every 3 weeks along with prednisone 5 mg/day, AU$ 161 per month) were obtained from the Pharmaceutical Benefits Scheme (items 11206T, 1935W, 10174L, and 10148D) (30-34). Costs for treatment of grade III adverse events were obtained from studies by Barqawi et al. (35) and Roeland et al. (36) and converted to 2021 Australian dollars using the Campbell and Cochrane Economics Methods Group (CCEMG) cost converter (37).

### Utilities

The utility weights for the progression-free state while receiving NHA (0.76; CI: 0.69–0.78) was based on findings for mCRPC patients receiving abiraterone therapy in the study by Clarke et al. (38). We assumed similar utility for olaparib treatment. Utility weights for docetaxel (0.69; CI: 0.59–0.80) and post-docetaxel (supportive care in the current model) (0.37; CI: 0.33–0.41) were obtained from studies by Lloyd et al. (39) and Barqawi et al. (35). Disutility weights of grade III adverse events (anemia, fatigue, vomiting, and back pain) were obtained from studies by Barqawi et al. (35) and Hall et al. (40). The values for utility weights in the aforementioned studies were derived from patient responses to the EuroQoL-5D (41;42).

### Analysis

The cost-effectiveness analysis was performed from the Australian health system perspective. We used monthly cycles to estimate the costs and outcomes were expressed in quality-adjusted life-years (QALYs) gained over a 5-year time horizon, typical of the mCRPC population (43-45). The incremental cost effectiveness ratio (ICER) was calculated and in line with Australian guidelines (46), costs and outcomes were discounted at an annual discount rate of 5 percent.

Decision uncertainty was characterized using probabilistic sensitivity analysis. Parameters were assigned plausible distributions, and a set of input parameter values were drawn by random sampling (10,000 iterations) from each distribution. Probabilistic sensitivity analysis outcomes were used to estimate value of information including the Expected Value of Perfect Information (EVPI) using the nonparametric regression approach (47;48). Additionally, several one-way sensitivity analyses adjusting for the spread of model parameters were performed. The range of probable values for each parameter was derived from reported values in the original resource article and where information was not available, we assumed a 20 percent change from base-case value. Apart from the base-case analysis which uses expected summary statistics for model parameters (Table 1), we also performed scenario analyses for varying *BRCA* prevalence (6.2 percent (4), 14.0 percent (22)), uptake of germline testing (49;50) and also explored the price threshold of olaparib to arrive at cost-effective findings. In the absence of an official cut-off for WTP in Australia, the National Institute of Health and Clinical Excellence (NICE) threshold for appraisal of life-extending, end of life treatments was used to serve as a guideline for a cost-effectiveness ceiling and the WTP was evaluated at AU$ 100,000/QALY (51).

The conceptual/face validity of the model was confirmed by experts, that is, practicing clinical oncologists. Validity of the computerized model was affirmed by the coauthors (H.T. and P.S.) who are experienced health economists. Additionally, Markov traces of the health states for *BRCA* positive patients, *BRCA* carriers and *BRCA* negative/noncarriers across the time horizon have been provided in Supplementary S2–S4.

Finally, a Consolidated Health Economic Evaluation Reporting Standards (CHEERS) checklist of the key items in the study was also performed and is available online.

## Results

**Figure 2.**
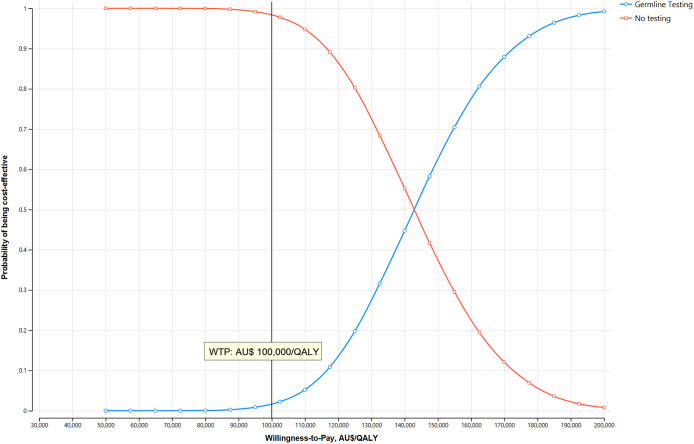
Cost-effectiveness acceptability curves for BRCA testing-guided therapy versus standard care in the base case model.

**Figure 3.**
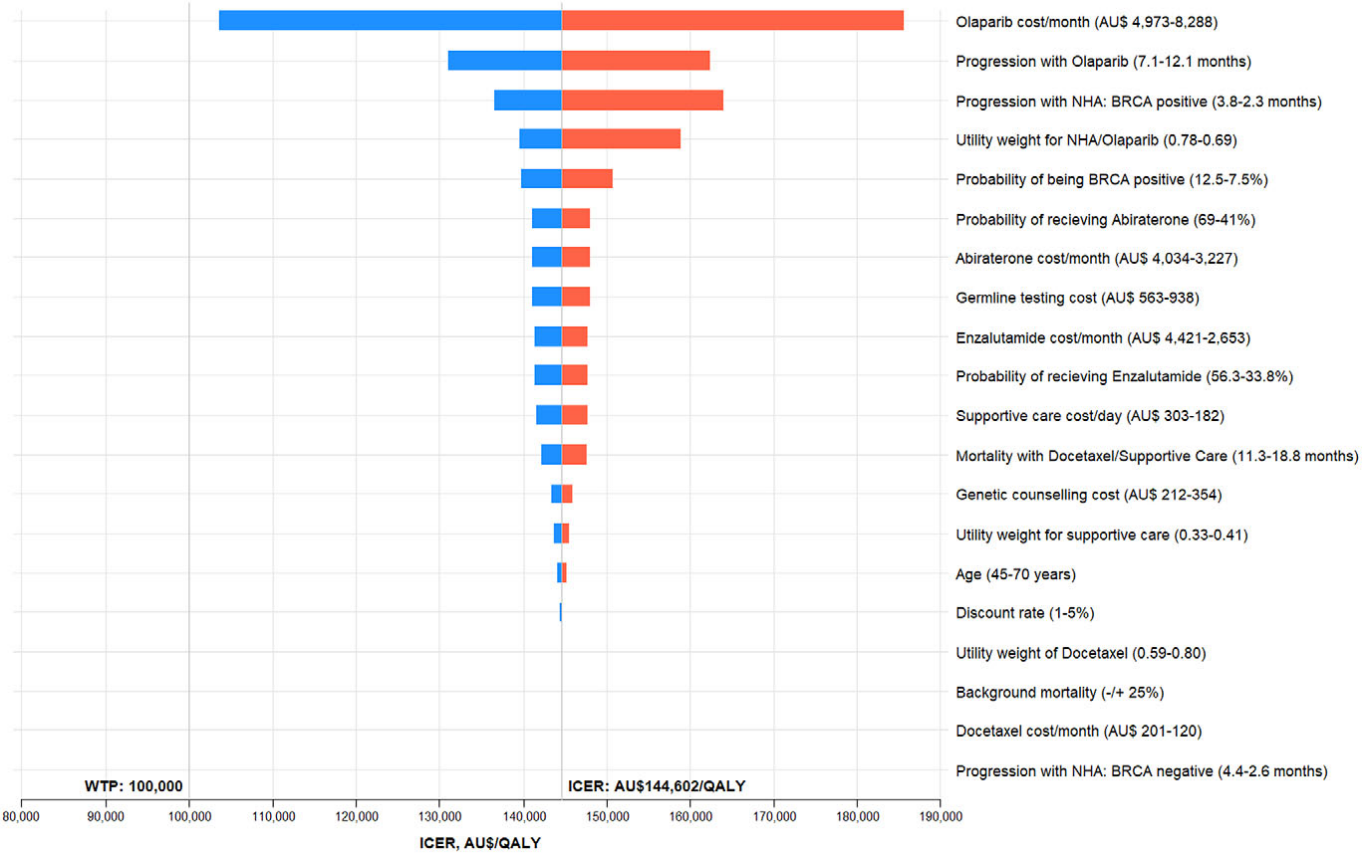
Tornado diagram of one-way sensitivity analyses of olaparib versus standard care in the base case analysis.

The results of the base-case analyses have been presented in Table 2. Compared to the standard care pathway, *BRCA* testing-guided olaparib treatment was associated with an additional cost of AU$ 7,841 and a gain of 0.06 QALYs. The resulting incremental cost-effectiveness ratio (ICER) of AU$ 143,613 per QALY for the *BRCA* testing pathway was substantially higher than the WTP threshold, suggesting that olaparib therapy was not cost-effective at its public price. At AU$ 100,000 WTP threshold, the probability that *BRCA* testing guided treatment is cost-effective was 1.7 percent. The cost-effectiveness acceptability curves, demonstrating the likelihood of germline testing being cost-effective, have been provided in Figure 2. The EVPI per person was AU$ 6.45 which is AU$ 138,255 for the population affected by the decision over 5 years, assuming the annual incidence of mCRPC is 4,286 patients each year. (52). The parameter contributing most to uncertainty was olaparib cost, with a population expected value of partially perfect information (EVPPI) of AU$ 88,169. The one-way sensitivity analysis (Figure 3) demonstrates that cost of olaparib, PFS during olaparib treatment, PFS with NHA in potential *BRCA* positive patients receiving standard care, the utility weights for NHA/olaparib treatment and the probability of being *BRCA* positive upon testing were the top five parameters influencing the ICER. The costs of adverse events (grade ≥ 3 or higher) in the cancer setting are substantial (53), yet they did not have a substantial impact on the results. There was a considerable shift in base case estimates over the range of plausible values for some variables (e.g., cost of olaparib), yet the ICERs remained higher than the WTP thresholds.

**Table 2.** Results of cost-effectiveness analysis

Strategy	Costs (discounted)	QALYs (discounted)	Incremental costs	Incremental QALYs	Incremental cost-effectiveness ratio	Optimal strategy in PSA
Base case						
Standard care, no *BRCA* testing	AU$ 103,335	0.90	Referent	Referent	Referent	98.28%*
*BRCA* testing and personalized Rx	AU$ 111,177	0.96	AU$ 7,841	0.06	AU$ 143,613/QALY	1.72%*
Scenario with 30% discount on olaparib cost						
Standard care, no *BRCA* testing	AU$ 103,335	0.90	Referent	Referent	Referent	33.93%*
*BRCA* testing and personalized Rx	AU$ 108,449	0.96	AU$ 5,113	0.06	AU$ 93,646/QALY	66.07%*
Scenario with 6.2% *BRCA* prevalence						
Standard care, no *BRCA* testing	AU$ 103,408	0.90	Referent	Referent	Referent	99.68%*
*BRCA* testing and personalized Rx	AU$ 108,662	0.93	AU$ 5,254	0.03	AU$ 155,211/QALY	0.32%*
Scenario with 14% *BRCA* prevalence						
Standard care, no *BRCA* testing	AU$ 103,259	0.90	Referent	Referent	Referent	96.86%*
*BRCA* testing and personalized Rx	AU$ 113,824	0.97	AU$ 10,565	0.07	AU$ 138,207/QALY	3.14%*
Scenario with 90% uptake in germline testing						
Standard care, no *BRCA* testing	AU$ 103.355	0.90	Referent	Referent	Referent	98.58%*
*BRCA* testing and personalized Rx	AU$ 109,814	0.94	AU$ 6,460	0.04	AU$ 144,990/QALY	1.42%*

* Willingness to pay (WTP): AU$ 100,000/QALY.

Abbreviations: PSA, probabilistic sensitivity analysis; QALYs, quality-adjusted life-years; Rx, treatment.

Supplementary analyses explored the cost-effectiveness of personalized treatment using *BRCA* prevalence rates of 6.2 percent (4) and 14.0 percent (22). Using the lower prevalence rate resulted in an ICER of AU$ 155,211/QALY, while the higher rate led to an ICER of AU$ 138,207/QALY. Uptake of germline testing in prostate cancer is usually high (90–95 percent) (49;50) and was therefore not considered in our base case analysis. However, if we were to the use the lower statistic among the two studies (49;50) (i.e., assume a 10 percent decline in *BRCA* testing), the resulting ICER of AU$ 144,990/QALY, was a marginal increase from our base case findings (ICER: AU$ 143,613/QALY).

We also examined the effects of applying additional discounts on olaparib pricing. As illustrated in Figure S5 in the Supplementary Material and Table 1, a 30 percent discount on olaparib cost was required to achieve an ICER below 100,000 (AU$ 93,646/QALY).

## Discussion

The economics of codependent technologies such as genetic testing to identify patients that respond effectively to personalized medication is an emerging field of research (54;55). In the current study, we examined the cost-effectiveness of germline *BRCA* test guided treatment with the PARP inhibitor olaparib in mCRPC patients compared to the standard care alternative without germline testing. Our results suggest that olaparib therapy could be beneficial with an increase of 0.06 QALYs over the comparator but was not cost-effective (ICER: AU$ 143,613/QALY; WTP: AU$ 100,000/QALY), unless the price of the drug was further discounted by 30 percent (i.e., from AU$ 6,631/month to $ 4,642/month).

Our findings are plausible and could be explained by several factors. To begin, our modeling approach is coincidentally similar to the NICE guidance for olaparib for previously treated *BRCA* mutation-positive hormone relapsed metastatic prostate cancer (56). In our analysis, the price of olaparib (34) was 87.5–91.8 percent higher than the current standard of care options (i.e., the two NHAs, enzalutamide (33) or abiraterone (30) and as indicated by our one-way sensitivity analyses, was associated with the highest variance in ICERs. Additionally, men treated with olaparib demonstrated higher PFS compared to NHA in the standard care pathway and in essence were on the more expensive treatment for a longer duration (9). Although it was assumed that they accrued health benefits as they remained at this stage of treatment, the balance between increased cost of treatment and its health benefits (QALYs), was not sufficient to tip the scale below the WTP threshold. The prevalence of tested pathogenic variants could have also influenced the results (13). The study by Su et al. (13) demonstrated that olaparib was cost-saving when administered to men with any one of fifteen pathogenic variants (100 percent of their cohort) but had high ICERs when evaluated in a smaller subset of people positive to three of the fifteen variants (65 percent of their cohort). In summary, cost of olaparib treatment, PFS with olaparib, the health utility of remaining in this stage of treatment and the probability of being variant positive were some of the major factors influencing the cost-effectiveness of olaparib treatment.

There are several differences both in the methodology and magnitude of results from our analyses and the earlier studies (12-14). First, all three previous studies relied heavily on data from the PROfound trial (9), where PFS with olaparib was compared against NHA in men who were variant positive. The co-dependence of olaparib therapy on the results of germline testing was not assessed, that is, the decision tree did not include men who were variant negative or those that received standard care without genetic testing. In contrast, we employed a more appropriate design and considered the cost-effectiveness of treatment pathways based on germline testing compared to a no testing approach. The importance of inclusion of price of genetic testing in appraising cost-effectiveness was emphasized by NICE in its evaluation report of olaparib (NICE TA 887) (56) Section 3.21, “The costs of testing BRCA mutations should be included in the cost-effectiveness estimates.” In Australia, testing to inform the eligibility for olaparib treatment is currently subsidized by the MBS, yet prices continue to remain substantial at AU$ 1,000. Second, there were also differences in the number of variants assessed during testing in our approach and the previous studies. While they (12-14) assessed three or fifteen pathogenic variants, we limited our analyses to germline testing of BRCA1/2, the pathogenic variants listed for subsidized treatment with olaparib in Australia (34). Not all pathogenetic variants associated with prostate cancer have the same mutagenic potential, that is, penetrance and a previous expert panel consensus recommendation has advocated for priority testing of *BRCA2/1* and DNA mismatch repair (MMR) genes over other variants (57). The PARP inhibitors such as olaparib are also more efficacious in treating patients with homologous double-stranded DNA-repair (58), the primary impairment mechanism of cancers associated with *BRCA* mutations (59), further justifying our strategy for *BRCA1/2* testing. Third, cross-resistance is known phenomenon between taxanes, NHAs, and PARP inhibitors (60;61), and the sequence of treatment among these classes of drugs could have important implications on their effectiveness (60;61). The lines of treatment in the economic modeling of previous studies was unclear (12;13). We adopt a more practice-based line of treatment (18;62) likely to minimize the effect of cross-resistance (63) (standard care: NHA > NHA > taxane > supportive care; *BRCA* positive: NHA > PARP > taxane > supportive care) and therefore provide an appropriate and replicable treatment scenario (25). Finally, we also explored thresholds at which further subsidization in olaparib pricing would sway results toward cost-effectiveness.

Our findings are not without limitations. Although we have strived to perform the analysis from an Australian health payer perspective, information for some of the parameters in the model was derived from studies based in the U.S. Second, we assumed that all patients were docetaxel naïve at the start of treatment (62). However, the inputs for PFS with olaparib or NHA in potential *BRCA* positive patients without testing were derived from the PROfound trial (9), where 65 percent of the patients received previous taxane therapy. Third, the comparator for PARP therapy in our analysis was repeat treatment with another NHA. Recent guidance suggests that this offers little benefit and patients with disease progression should ideally be treated with a taxane (docetaxel or cabazitaxel) or receive basic supportive care (56). Fourth, we would like to acknowledge that the health state utilities were captured from multiple studies across different settings and as such may not be completely appropriate for the intended cohort of mCRPC patients. Finally, we acknowledge that olaparib could have received additional subsidies through commercial arrangements. However, due to the unavailability of this special pricing the current evaluation was based on the market price of olaparib. The differences in our assumptions and those from our resource data do lend some uncertainty to our results. Yet we have strived to utilize the most pertinent available information and attribute discrepancies in assumptions among resource data and our test case to the paucity of statistics within the literature.

The PARP inhibitors have significant survival benefits (9;64) and are approved (23;65) groundbreaking treatments for prostate cancer. Yet, there is a lack of clarity about the ideal target population for their use in the prostate cancer disease spectrum (58). The variability in effectiveness based on pathogenic variants (57), modeling differences (partitioned versus state transition models) (15) and the failure to account for the codependent nature of PARP inhibitor therapy based on the results of genetic testing may explain the inconsistencies in findings from previous economic studies (12-14). In the current evaluation, we have assessed the cost-effectiveness of germline testing and olaparib as codependent technologies. Our findings suggest that from an Australian health system perspective, second line treatment with olaparib in mCRPC may be potentially cost-effective if the current market price of the drug is reduced by 30 percent.

## Supporting information

Teppala et al. supplementary material 1 Teppala et al. supplementary material

Teppala et al. supplementary material 2 Teppala et al. supplementary material

## Data Availability

Data sharing is not applicable to this article as no datasets were generated during the current study.

## Supplementary material

The supplementary material for this article can be found at https://doi.org/10.1017/S0266462324000011.

## Key points

- Poly-adenosine diphosphate ribose polymerase (PARP) inhibitors, such as olaparib target repair pathways in cancer cells and are breakthrough therapies in the management of metastatic castration resistant prostate cancer (mCRPC). The cost-effectiveness of olaparib, however, is not clear.
- Economic evaluations of olaparib have overlooked the need for genetic testing to appropriately target treatment. This paper is the first evaluation of codependent technologies (germline testing and olaparib therapy) in mCRPC from Australia.
- Our evaluation suggests that germline *BRCA* testing-guided treatment with olaparib in mCRPC may be cost-effective after applying a 30 percent reduction to the existing market price of the drug.

## References

[1] AIHW. Cancer data in Australia. 2022. Available from: https://www.aihw.gov.au/reports/cancer/cancer-data-in-australia/contents/about.

[2] Giri VN, Hyatt C, Gomella LG. Germline testing for men with prostate cancer: Navigating an expanding new world of genetic evaluation for precision therapy and precision management. J Clin Oncol. 2019;37:1455–1459. 10.1200/jco.18.02181. 30978156

[3] Nicolosi P, Ledet E, Yang S, et al. Prevalence of germline variants in prostate cancer and implications for current genetic testing guidelines. JAMA Oncol. 2019;5:523–528. 10.1001/jamaoncol.2018.6760. 30730552 PMC6459112

[4] Pritchard CC, Mateo J, Walsh MF, et al. Inherited DNA-repair gene mutations in men with metastatic prostate cancer. N Engl J Med. 2016;375:443–453. 10.1056/NEJMoa1603144. 27433846 PMC4986616

[5] The Cancer Genome Atlas Research Network. The molecular taxonomy of primary prostate cancer. Cell. 2015;163:1011–1025. 10.1016/j.cell.2015.10.025. 26544944 PMC4695400

[6] Castro E, Goh C, Olmos D, et al. Germline BRCA mutations are associated with higher risk of nodal involvement, distant metastasis, and poor survival outcomes in prostate cancer. J Clin Oncol. 2013;31:1748–1757. 10.1200/jco.2012.43.1882. 23569316 PMC3641696

[7] Gallagher DJ, Gaudet MM, Pal P, et al. Germline BRCA mutations denote a clinicopathologic subset of prostate cancer. Clin Cancer Res. 2010;16:2115–2121. 10.1158/1078-0432.Ccr-09-2871. 20215531 PMC3713614

[8] Rose M, Burgess JT, O’Byrne K, Richard DJ, Bolderson E. PARP inhibitors: Clinical relevance, mechanisms of action and tumor resistance. Front Cell Dev Biol. 2020;8:564601. 10.3389/fcell.2020.564601. 33015058 PMC7509090

[9] de Bono J, Mateo J, Fizazi K, et al. Olaparib for metastatic castration-resistant prostate cancer. N Engl J Med. 2020;382:2091–2102. 10.1056/NEJMoa1911440. 32343890

[10] Abida W, Patnaik A, Campbell D, et al. Rucaparib in men with metastatic castration-resistant prostate cancer Harboring a BRCA1 or BRCA2 gene alteration. J Clin Oncol. 2020;38:3763–3772. 10.1200/jco.20.01035. 32795228 PMC7655021

[11] TGA. Department of Health and Aged Care: Therapeutic Goods Administration. Products we regulate. 2022. Available from: https://www.tga.gov.au/products.

[12] Li Y, Lin S, Zhong L, et al. Is olaparib cost effective in metastatic castration-resistant prostate cancer patients with at least one favorable gene mutation in BRCA1, BRCA2 or ATM? Pharmacogenomics. 2021;22:809–819. 10.2217/pgs-2021-0061. 34517749

[13] Su D, Wu B, Shi L. Cost-effectiveness of genomic test-directed olaparib for metastatic castration-resistant prostate cancer. Front Pharmacol. 2021;11:2436. 10.3389/fphar.2020.610601 PMC7870786 33574757

[14] Xu C, Cai J, Zhuang J, et al. Cost-effectiveness of olaparib, a PARP inhibitor, for patients with metastatic castration-resistant prostate cancer in China and United States. Ann Transl Med. 2022;10:830. 10.21037/atm-22-3637. 36034977 PMC9403933

[15] Woods BS, Sideris E, Palmer S, Latimer N, Soares M. Partitioned survival and state transition models for healthcare decision making in oncology: Where are we now? Value Health. 2020;23:1613–1621. 10.1016/j.jval.2020.08.2094. 33248517

[16] George DJ, Sartor O, Miller K, et al. Treatment patterns and outcomes in patients with metastatic castration-resistant prostate cancer in a real-world clinical practice setting in the United States. Clin Genitourin Cancer. 2020;18:284–294. 10.1016/j.clgc.2019.12.019. 32057714

[17] TreeAge. TreeAge pro healthcare 2023. Williamstown, MA: TreeAge Software; 2022. Available from: http://www.treeage.com.

[18] NCCN. Clinical practice guidelines in oncology, prostate cancer. Version 4. 2022. Available from: https://www.nccn.org/professionals/physician_gls/pdf/prostate.pdf.

[19] Pollard ME, Moskowitz AJ, Diefenbach MA, Hall SJ. Cost-effectiveness analysis of treatments for metastatic castration resistant prostate cancer. Asian J Urol. 2017;4:37–43. 10.1016/j.ajur.2016.11.005. 29264205 PMC5730904

[20] Tannock IF, de Wit R, Berry WR, et al. Docetaxel plus prednisone or mitoxantrone plus prednisone for advanced prostate cancer. N Engl J Med. 2004;351:1502–1512. 10.1056/NEJMoa040720. 15470213

[21] Parker C, Castro E, Fizazi K, et al. Prostate cancer: ESMO clinical practice guidelines for diagnosis, treatment and follow-up. Ann Oncol. 2020;31:1119–1134. 10.1016/j.annonc.2020.06.011. 32593798

[22] Robinson D, Van Allen EM, Wu YM, et al. Integrative clinical genomics of advanced prostate cancer. Cell. 2015;161:1215–1228. 10.1016/j.cell.2015.05.001. 26000489 PMC4484602

[23] MSAC. Testing of tumour prostate tissue to detect BRCA1/2 pathogenic gene variants in men with metastatic castration-resistant prostate cancer to help determine eligibility for PBS olaparib. 2021. Available from: http://www.msac.gov.au/internet/msac/publishing.nsf/Content/1618-public.

[24] Hussain M, Mateo J, Fizazi K, et al. Survival with olaparib in metastatic castration-resistant prostate cancer. N Engl J Med. 2020;383:2345–2357. 10.1056/NEJMoa2022485. 32955174

[25] Shore ND, Laliberté F, Ionescu-Ittu R, et al. Real-world treatment patterns and overall survival of patients with metastatic castration-resistant prostate cancer in the US prior to PARP inhibitors. Adv Ther. 2021;38:4520–4540. 10.1007/s12325-021-01823-6. 34282527 PMC8342357

[26] ABS. Life tab 2018–2020. 2021. Available from: https://www.abs.gov.au/statistics/people/population/life-tables/2018-2020.

[27] Miyake H, Sato R, Watanabe K, et al. Prognostic significance of third-line treatment for patients with metastatic castration-resistant prostate cancer: Comparative assessments between cabazitaxel and other agents. Int J Clin Oncol. 2021;26:1745–1751. 10.1007/s10147-021-01956-2. 34255227

[28] MBS. Category 6: Pathology services. Detection of germline BRCA 1 or BRCA2 pathogenic gene variants. Item 73304. 2022. Available from: http://www9.health.gov.au/mbs/fullDisplay.cfm?type=item&q=73304&qt=item&criteria=73304.

[29] MBS. Category 1: Professional attendances. Consultant physician attendaces. Genetic counsellor consultation. Item 132. 2022. Available from: http://www9.health.gov.au/mbs/fullDisplay.cfm?type=item&q=132&qt=item&criteria=132.

[30] PBS. General schedule. Antineoplastic and immunomodulating agents. Abiraterone. Item 11206T. 2022. Available from: https://www.pbs.gov.au/medicine/item/11206T.

[31] PBS. General schedule. Systemic hormonal preperations. Prednisone. Item 1935W. 2022. Available from: https://www.pbs.gov.au/medicine/item/1935W.

[32] PBS. General schedule. Antineoplastic and immunomodulating agents. Docetaxel. Item 10148D. 2022. Available from: https://www.pbs.gov.au/medicine/item/10148D-10158P.

[33] PBS. General schedule. Antineoplastic and immunomodulating agents. Enzalutamide. Item 10174L. 2022. Available from: https://www.pbs.gov.au/medicine/item/10174L.

[34] PBS. General schedule. Antineoplastic and immunomodulating agents. Olaparib. Item 11528R. 2022. Available from: https://www.pbs.gov.au/medicine/item/11528R-11539H-12157W-12161C-12913P-12929L.

[35] Barqawi YK, Borrego ME, Roberts MH, Abraham I. Cost-effectiveness model of abiraterone plus prednisone, cabazitaxel plus prednisone and enzalutamide for visceral metastatic castration resistant prostate cancer therapy after docetaxel therapy resistance. J Med Econ. 2019;22:1202–1209. 10.1080/13696998.2019.1661581. 31452414

[36] Roeland E, Nipp RD, Ruddy KJ, et al. Inpatient hospitalization costs associated with nausea and vomiting among patients with cancer. J Clin Oncol. 2018;36:112–112. 10.1200/JCO.2018.36.34_suppl.112.

[37] CCEMG-EPPI-Centre. Campbell and cochrane economics methods group and the evidence for policy and practice information and coordinating centre. CCEMG–EPPI-centre cost converter. 2022. Available from: https://eppi.ioe.ac.uk/costconversion/.

[38] Clarke CS, Hunter RM, Gabrio A, et al. Cost-utility analysis of adding abiraterone acetate plus prednisone/prednisolone to long-term hormone therapy in newly diagnosed advanced prostate cancer in England: Lifetime decision model based on STAMPEDE trial data. PLoS One. 2022;17:e0269192. 10.1371/journal.pone.0269192. 35653395 PMC9162346

[39] Lloyd AJ, Kerr C, Penton J, Knerer G. Health-related quality of life and health utilities in metastatic castrate-resistant prostate cancer: A survey capturing experiences from a diverse sample of UK patients. Value Health. 2015;18:1152–1157. 10.1016/j.jval.2015.08.012. 26686802

[40] Hall F, de Freitas HM, Kerr C, et al. Estimating utilities/disutilities for high-risk metastatic hormone-sensitive prostate cancer (mHSPC) and treatment-related adverse events. Qual Life Res. 2019;28:1191–1199. 10.1007/s11136-019-02117-9. 30767088 PMC6470112

[41] EuroQoL. About EuroQoL-5D-3L. 2021. Available from: https://euroqol.org/eq-5d-instruments/eq-5d-3l-about/.

[42] Al-Batran SE, Hozaeel W, Tauchert FK, et al. The impact of docetaxel-related toxicities on health-related quality of life in patients with metastatic cancer (QoliTax). Ann Oncol. 2015;26:1244–1248. 10.1093/annonc/mdv129. 25755108

[43] Aly M, Leval A, Schain F, et al. Survival in patients diagnosed with castration-resistant prostate cancer: A population-based observational study in Sweden. Scand J Urol. 2020;54:115–121. 10.1080/21681805.2020.1739139. 32266854

[44] Armstrong AJ, Lin P, Tombal B, et al. Five-year survival prediction and safety outcomes with enzalutamide in men with chemotherapy-naïve metastatic castration-resistant prostate cancer from the PREVAIL trial. Eur Urol. 2020;78:347–357. 10.1016/j.eururo.2020.04.061. 32527692

[45] Modonutti D, Majdalany SE, Corsi N, et al. A novel prognostic model predicting overall survival in patients with metastatic castration-resistant prostate cancer receiving standard chemotherapy: A multi-trial cohort analysis. Prostate. 2022;82:1293–1303. 10.1002/pros.24403. 35790016

[46] PBAC. Guidelines for preparing submissions to the Pharmaceutical Benefits Advisory Committee. 2016. Available from: https://pbac.pbs.gov.au/.

[47] Strong M, Oakley JE, Brennan A. Estimating multiparameter partial expected value of perfect information from a probabilistic sensitivity analysis sample: A nonparametric regression approach. Med Decis Mak. 2014;34:311–326. 10.1177/0272989x13505910. PMC4819801 24246566

[48] Tuffaha HW, Strong M, Gordon LG, Scuffham PA. Efficient value of information calculation using a nonparametric regression approach: An applied perspective. Value Health. 2016;19:505–509. 10.1016/j.jval.2016.01.011. 27325343

[49] Hamilton JG, Symecko H, Spielman K, et al. Uptake and acceptability of a mainstreaming model of hereditary cancer multigene panel testing among patients with ovarian, pancreatic, and prostate cancer. Genet Med. 2021;23:2105–2113. 10.1038/s41436-021-01262-2. 34257420 PMC8556289

[50] Scheinberg T, Goodwin A, Ip E, et al. Evaluation of a mainstream model of genetic testing for men with prostate cancer. JCO Oncol Pract. 2021;17:e204–e216. 10.1200/op.20.00399. 32970524

[51] NICE. National Institute for Health and Clinical Excellence. Appraising life-extending, end of life treatments. 2009). Available from: https://www.nice.org.uk/guidance/gid-tag387/documents/appraising-life-extending-end-of-life-treatments-paper2#:~:text=2%20Criteria%20for%20appraisal%20of%20end%20of%20life%20treatments&text=24%20months%20and%3B-,2.1.,indicated%2C%20for%20small%20patient%20populations.

[52] AIHW. Prostate cancer in Australia statistics. 2022. Available from: https://www.canceraustralia.gov.au/cancer-types/prostate-cancer/statistics.

[53] Wong W, Yim YM, Kim A, et al. Assessment of costs associated with adverse events in patients with cancer. PLoS One. 2018;13:e0196007. 10.1371/journal.pone.0196007. 29652926 PMC5898735

[54] PBAC. Pharmaceutical Benefits Advisory Committee. Codependent technologies. 2016. Available from: https://pbac.pbs.gov.au/product-type-4-codependent-technologies.html.

[55] Merlin T, Farah C, Schubert C, et al. Assessing personalized medicines in Australia: A national framework for reviewing codependent technologies. Med Decis Mak. 2013;33:333–342. 10.1177/0272989x12452341. PMC3757917 22895559

[56] NICE. Olaparib for previously treated BRCA mutation-positive hormone-relapsed metastatic prostate cancer [TA887]. 2023. Available from: https://www.nice.org.uk/guidance/ta887/chapter/3-Committee-discussion. 40138501

[57] Giri VN, Knudsen KE, Kelly WK, et al. Implementation of germline testing for prostate cancer: Philadelphia prostate cancer consensus conference 2019. J Clin Oncol. 2020;38:2798–2811. 10.1200/jco.20.00046. 32516092 PMC7430215

[58] Antonarakis ES, Gomella LG, Petrylak DP. When and how to use PARP inhibitors in prostate cancer: A systematic review of the literature with an update on on-going trials. Eur Urol Oncol. 2020;3:594–611. 10.1016/j.euo.2020.07.005. 32814685

[59] Roy R, Chun J, Powell SN. BRCA1 and BRCA2: Different roles in a common pathway of genome protection. Nat Rev Cancer. 2011;12:68–78. 10.1038/nrc3181. 22193408 PMC4972490

[60] Lombard AP, Liu C, Armstrong CM, et al. Overexpressed ABCB1 induces olaparib-taxane cross-resistance in advanced prostate cancer. Transl Oncol. 2019;12:871–878. 10.1016/j.tranon.2019.04.007. 31075528 PMC6510951

[61] van Soest RJ, van Royen ME, de Morrée ES, et al. Cross-resistance between taxanes and new hormonal agents abiraterone and enzalutamide may affect drug sequence choices in metastatic castration-resistant prostate cancer. Eur J Cancer. 2013;49:3821–3830. 10.1016/j.ejca.2013.09.026. 24200698

[62] Cornford P, van den Bergh RCN, Briers E, et al. EAU-EANM-ESTRO-ESUR-SIOG guidelines on prostate cancer. Part II-2020 update: Treatment of relapsing and metastatic prostate cancer. Eur Urol. 2021;79:263–282. 10.1016/j.eururo.2020.09.046. 33039206

[63] Maurice Dror C, Chi KN, Khalaf DJ. Finding the optimal treatment sequence in metastatic castration-resistant prostate cancer-a narrative review. Transl Androl Urol. 2021;10:3931–3945. 10.21037/tau-20-1341. 34804836 PMC8575566

[64] Mateo J, Carreira S, Sandhu S, et al. DNA-repair defects and olaparib in metastatic prostate cancer. N Engl J Med. 2015;373:1697–1708. 10.1056/NEJMoa1506859. 26510020 PMC5228595

[65] Helleday T. PARP inhibitor receives FDA breakthrough therapy designation in castration resistant prostate cancer: Beyond germline BRCA mutations. Ann Oncol. 2016;27:755–757. 10.1093/annonc/mdw048. 26865580

[66] Lee JH, Kim SH, Oh SY, et al. Third-line docetaxel chemotherapy for recurrent and metastatic gastric cancer. Korean J Intern Med. 2013;28:314–321. 10.3904/kjim.2013.28.3.314. 23682225 PMC3654129

[67] Cronin P, Kirkbride B, Bang A, et al. Long-term health care costs for patients with prostate cancer: A population-wide longitudinal study in New South Wales, Australia. *Asia Pac J Clin Oncol.* 2017;13:160–171. 10.1111/ajco.12582. 27619777

