# Supplementary material for: The cost-effectiveness of germline BRCA testing-guided olaparib treatment in metastatic castration resistant prostate cancer
Source: Int J Technol Assess Health Care. 2024 Mar 5;40(1):e14. doi: 10.1017/S0266462324000011 (PMC11570197; doi:10.1017/S0266462324000011)
Supplement: Teppala et al. supplementary material 2 — Teppala et al. supplementary material [file S0266462324000011sup002.docx]

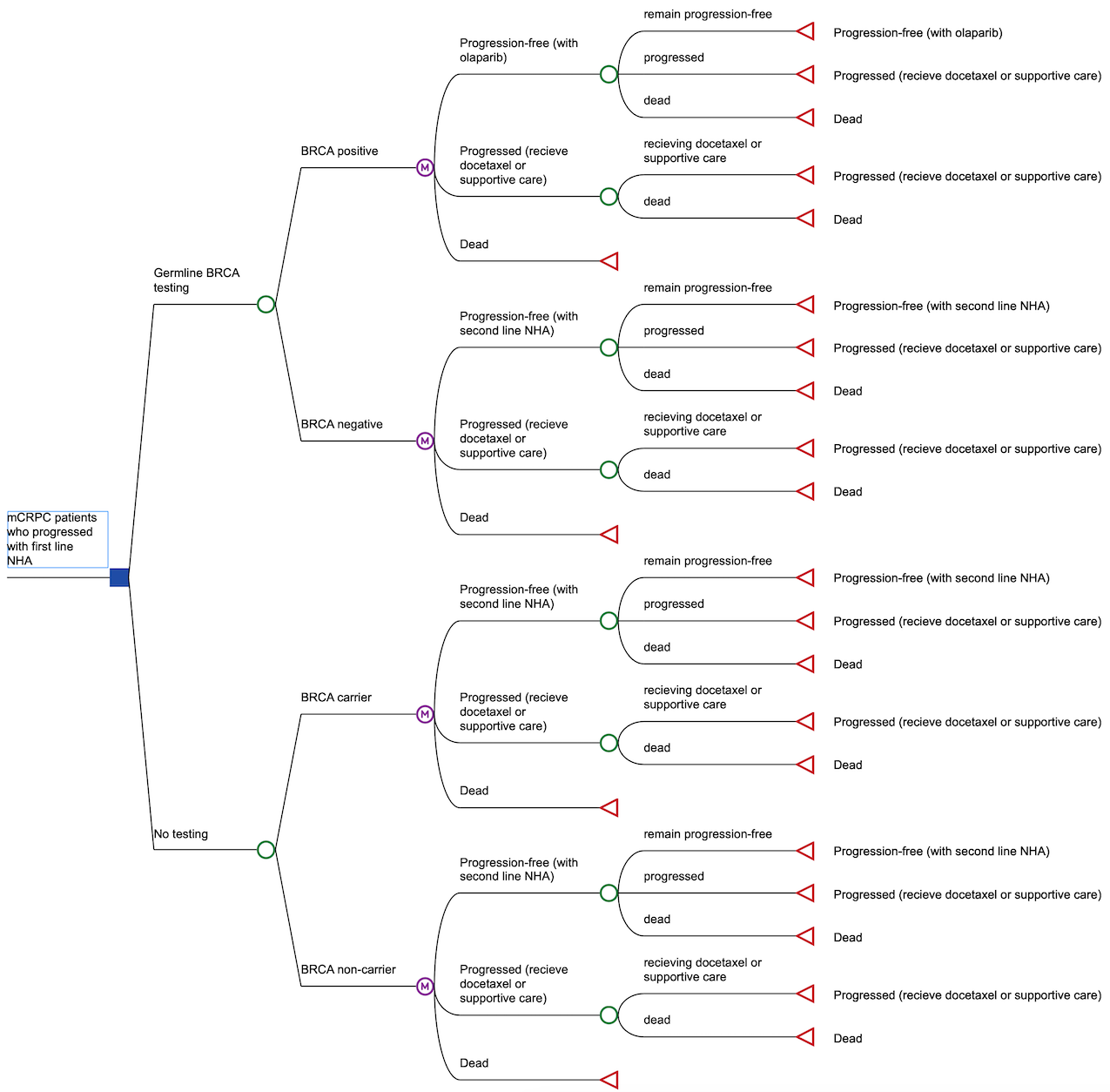


**Figure S1. Detailed schematic of the model**


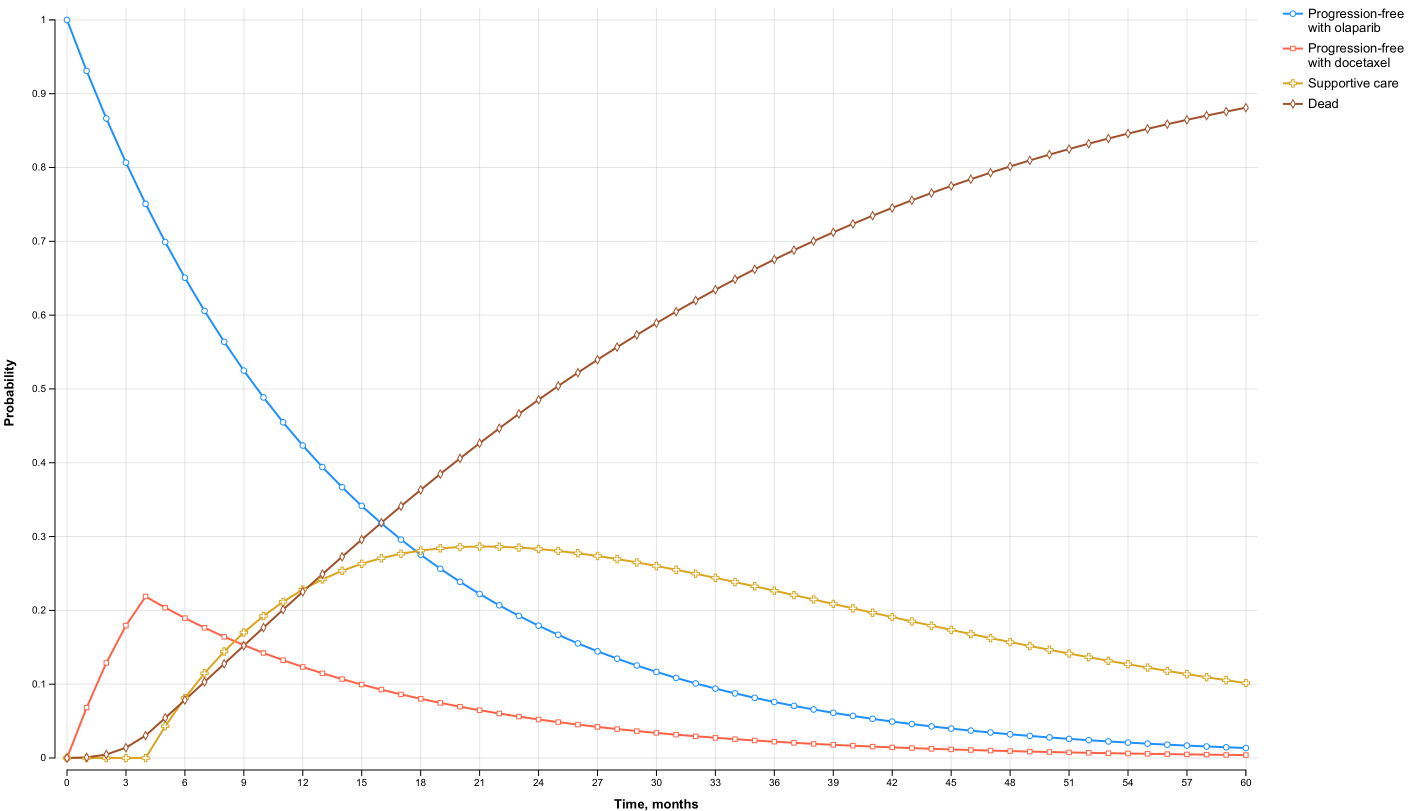


**Figure S2. Markov traces for BRCA positive patients receiving olaparib treatment**

**
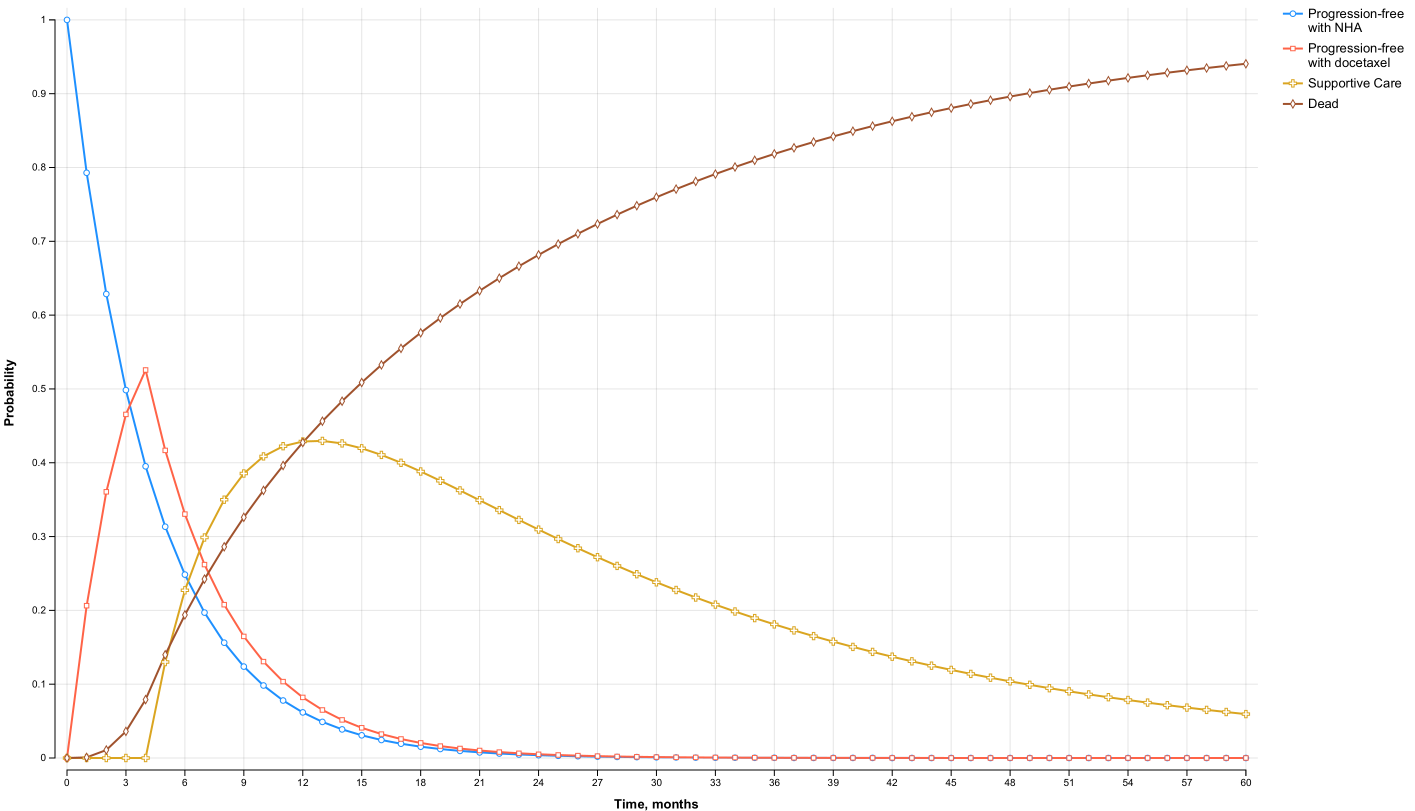
**

**Figure S3. Markov traces for BRCA carriers receiving NHA treatment**


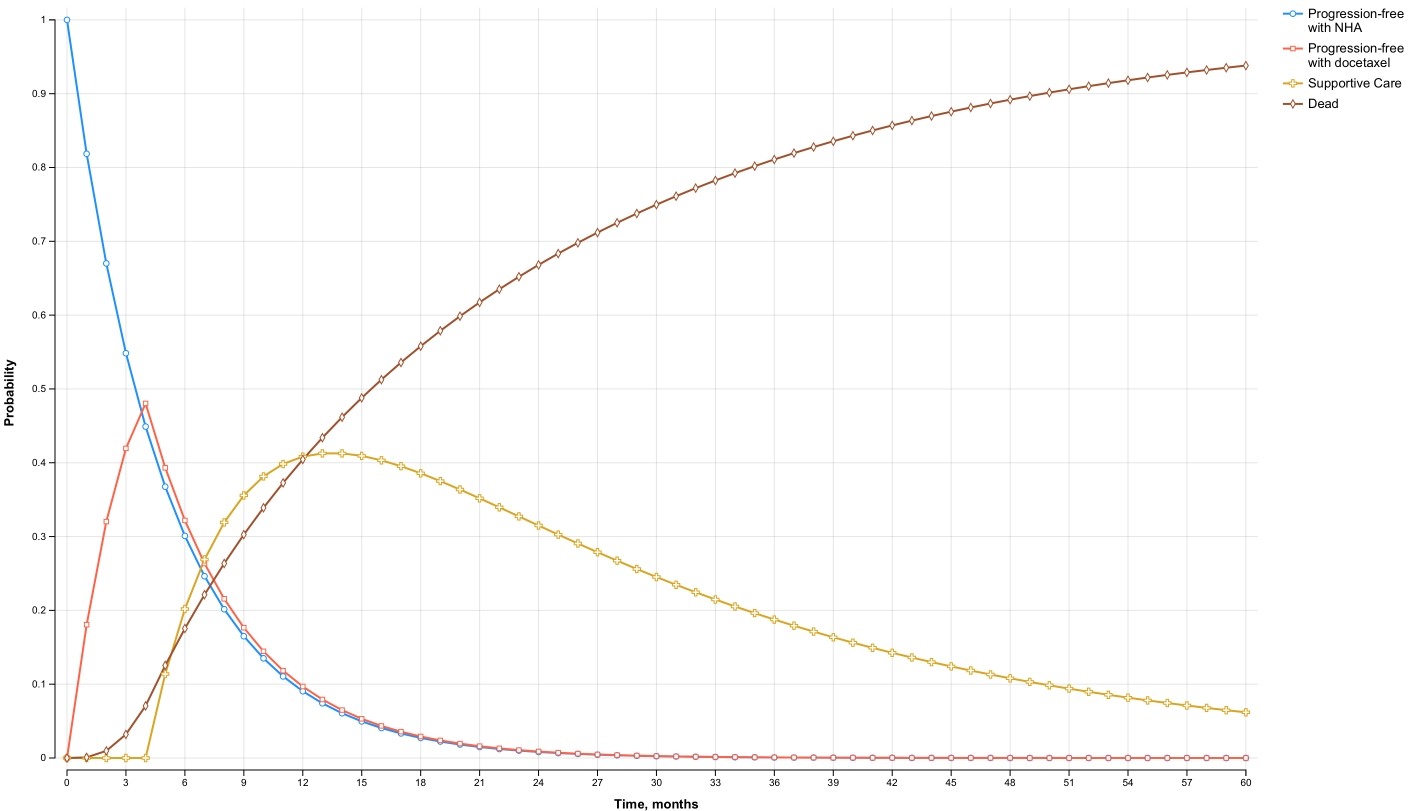


**Figure S4. Markov traces for BRCA negative/non-carriers receiving NHA treatment**

**Figure S5. Change in incremental cost-effectiveness ratio (ICER) with reduction in olaparib cost.**
